# Therapeutic potential of human breast milk-derived exosomes in necrotizing enterocolitis

**DOI:** 10.1186/s10020-024-01010-7

**Published:** 2024-12-19

**Authors:** Si-Jia Di, Xue-wei Cui, Tian-Jing Liu, Yong-Yan Shi

**Affiliations:** Department of Pediatrics, Shengjing Hospital of China Medical University, Shenyang, 110004 China

**Keywords:** Exosome, Human breast milk-derived exosome, Necrotizing enterocolitis, Neonate

## Abstract

Necrotizing enterocolitis (NEC) is a severe inflammatory and necrotizing disease of the intestine that primarily affects the neonates, particularly premature infants. It has a high incidence of approximately 8.9% in extremely preterm infants, with a mortality rate ranging from 20 to 30%. In recent years, exosomes, particularly those derived from breast milk, have emerged as potential candidates for NEC therapy. Human breast milk-derived exosomes (BME) have been shown to enhance intestinal barrier function, protect intestinal epithelial cells from oxidative stress, promote the proliferation and migration of intestinal epithelial cells, and reduce the severity of experimental NEC models. As a subset of extracellular vesicles, BME possess the membrane structure, low immunogenicity, and high permeability, making them ideal vehicles for the treatment of NEC. Additionally, exosomes derived from various sources, including stem cells, intestinal epithelial cells, plants, and bacteria, have been implicated in the development and protection of intestinal diseases. This article summarizes the mechanisms through which exosomes, particularly BME, exert their effects on NEC and discusses the feasibility and obstacles associated with this novel therapeutic strategy.

## Background

Necrotizing Enterocolitis (NEC) is a destructive gastrointestinal inflammatory disease that affects the newborns, particularly those with low birth weights. It is considered to be the most common and severe disorder in the neonatal intensive care unit (NICU). NEC has a high incidence of 8.9% in premature infants born at the gestational age of 22–28 weeks with a mortality rate of 20–30% (Meister et al. 2020). The pathophysiology of NEC involves a complex interplay of factors, including intestinal mucosal hypoxia and ischemic injury, microbial dysbiosis, premature birth, immature immune system, and formula feeding (Cai et al. 2022). Currently, the primary therapeutic approaches to NEC include cessation of feeding, antibiotic therapy, and surgical intervention; however, these measures have not significantly improved patient outcomes (Annette Gawron et al. 2024). Therefore, there is an urgent need for advanced strategies to prevent and treat NEC in preterm infants.

Breastfeeding has been demonstrated to lower the risk of NEC compared to formula feeding (Peng et al. 2022; Kathyayini et al. 2019). Recent studies have shown that breast milk derived-exosomes (BME) may possess potential therapeutic effects in reducing the incidence and severity of NEC (Pisano et al. 2020; He et al. 2021). Exosomes are natural vehicles for cell-to-cell communication, and their characteristics, including low immunogenicity, high permeability, stability in body fluids and intestinal protection make them an attractive option for NEC therapy (Manchon et al. 2021; Ghosh et al. 2020; Filip 2021). Herein, current studies on BME and their potential roles in NEC therapy are reviewed to delineate a new landscape for the treatment of NEC.

## Exosomes

**Fig. 1:**
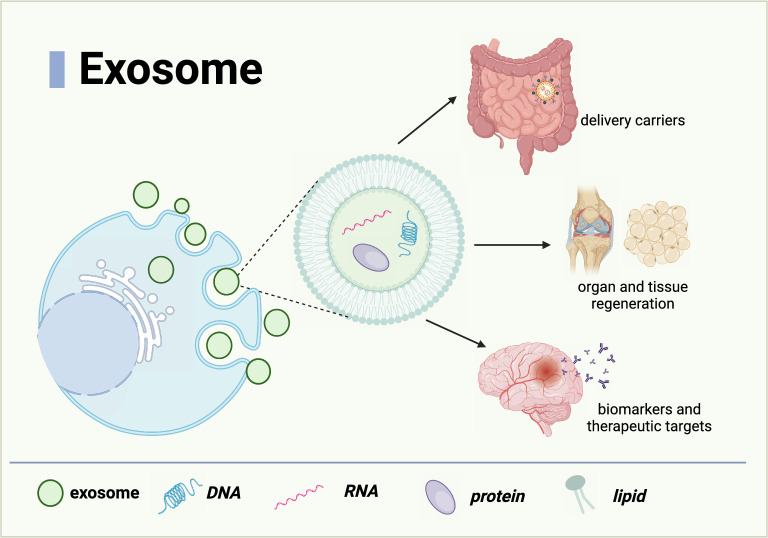
The composition and main application fields of exosomes. Multiple vesicles formed by endosomal invagination are released into the extracellular matrix, ultimately forming exosomes. Exosomes contain various components, mainly including proteins, nucleic acids, and lipids. Owing to their general characteristics, exosomes may be used as therapeutic agents. (Figure created with BioRender.com)

Multiple vesicles formed by endosomal invagination are released into the extracellular matrix via cell fusion, which eventually form exosomes. Exosomes are coated by a lipid bilayer membrane with a diameter of 30–150 nm (Doyle and Wang 2019; Lin et al. 2020). Exosomes usually contain proteins (such as heat shock proteins, tetraspanins, and tumor-specific glycoproteins) (Rastogi et al. 2021), nucleic acids (such as DNA, lncRNA, mRNA, and miRNA) (Su et al. 2022) or lipids (such as sphingomyelins, free fatty acids, and cholesterol) (Biadglegne et al. 2022). Exosomes are able to convey their cargo to target cells through body fluids including blood, saliva, cerebrospinal fluid, urine, bronchoalveolar lavage fluid and milk (Stephanie and Suresh2015). Once delivered to the target cells, exosomes can regulate the function and morphology of the recipient cells by activating different signaling pathways depending on the origins of exosomes and the state of the cells. Exosomes may function as biomarkers of pathological conditions (Jafari et al. 2022), delivery carriers (Pomatto et al. 2019), regulators of organ and tissue regeneration (Yu and Huang 2022), and therapeutic targets (Wang et al. 2019a) through these contents (Fig. 1).

Exosomes distribute in various cells and tissues of the body, and can also be found in plants and bacteria (Chen et al. 2020). The function of exosomes is partially related to their origins and compositions (Park et al. 2022; Matsuzaka and Yashiro 2022). Exosomes from breast milk, intestinal epithelial cells and plants are beneficial for the gut (Pisano et al. 2020; Yang et al. 2022; Hu et al. 2023); however, exosomes derived from bacteria and polymorphonuclear neutrophils promote the development of intestinal diseases and aggravate intestinal epithelial damage (Chen et al. 2021a; Engevik et al. 2021). Therefore, understanding the exact mechanisms by which the exosomes are involved in the development of NEC, as well as their impact on NEC morbidity and mortality, is important for future studies.

Furthermore, exosomes work as media for intracellular communication and pathological process. Recent studies have shown that BME can tolerate digestion in the stomach and pancreas, retain microRNA (miR) cargo, and may potentially be used as an oral delivery vehicle for drugs that are traditionally administered intravenously (Betker et al. 2019; Kahn et al. 2018). Consequently, the secretion, uptake, isolation, composition, function, and delivery mechanisms of BME have become new focus areas.

## Human breast milk-derived exosomes (BME)

Components in breast milk, including oligosaccharides, lactoferrin, growth factors, peptides, and other bioactive substances may help reduce NEC mortality (Nolan et al. 2019). BME are primarily produced by mammary epithelial cells. These cells are located in the mammary alveoli and are responsible for the synthesis and secretion of milk, which makes BME signaling molecules between mothers and infants (Bodo 2017). BME play an important role not only in nutritional provision but also in shaping the infant's immune system and gut development (Elif et al. 2024).

Although exosomes derived from breast milk were originally extracted and identified from human colostrum (Admyre et al. 1950), they can also be obtained from porcine, bovine, rat, and panda, and exhibit interspecies tolerance. Multiple sources of BME are known to bolster intestinal development to treat NEC (Xie et al. 2020; Li et al. 2019; Hock et al. 2017; Ma et al. 2017). The exosomal components in human breast milk can interact with a variety of cells and exert a wide range of biological effects. Human intestinal cells can take up BME to modulate the immune system and the maturation of neonatal gastrointestinal tract (Yalin et al. 2017; Sarah et al. 2018). Besides, BME may act on epithelial cells, cancer cells, immune cells, and other stromal cells to take part in the pathogenesis of diseases such as bronchopulmonary dysplasia, cancer, diabetes and human immunodeficiency virus infection (Zhou et al. 2022; Dazhi et al. 2023; Shah et al. 2022; Zahoor et al. 2020).

## Role of BME in NEC

**Fig. 2:**
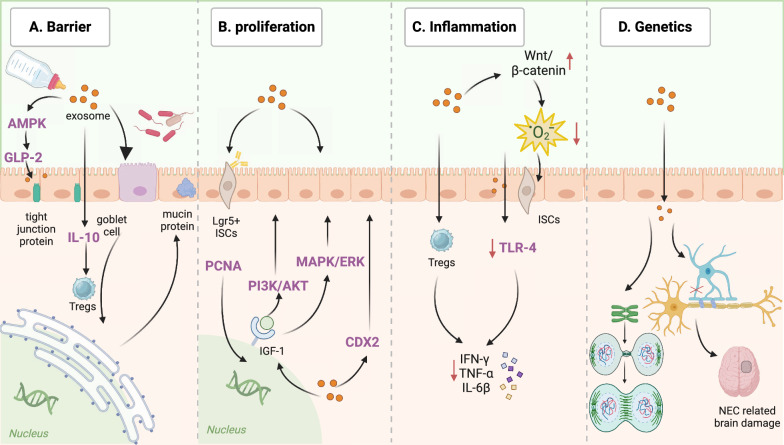
The therapeutic mechanisms of BME in NEC. Human breast milk-derived exosomes (BME) exert therapeutic effects on NEC at multiple levels, including the intestinal barrier, cell proliferation, inflammatory damage, genetics and metabolism. (Figure created with BioRender.com)

Research about breast milk has focused on BME and suggested their protective effects and the potential for the prevention and treatment in NEC models (O'Reilly et al. 2021). BME can modulate immune responses, promote intestinal health, reduce inflammation and regulate epigenetics (Hu et al. 2023; Pacella et al. 2023; Tong et al. 2023; Chen et al. 2016; Miyake et al. 2020; Dong et al. 2020). The specific therapeutic mechanisms of BME in terms of NEC treatment is discussed below (Fig. 2).

### Regulation of immunity

Intestinal mucosal immune dysregulation can be observed in NEC patients (Liu et al. 2023). BME can interact with immune cells, including macrophages and neutrophils, to stimulate the production of interleukin (IL)-10, thereby promoting the expansion of regulatory T cells (Tregs) by facilitating the differentiation of naïve CD4^+^ T cells into more Tregs (Pacella et al. 2023; Admyre et al. 2007). miR-155 that presents in BME has also been shown to promote thymic Tregs maturity and stability (Hata et al. 2010a; Melnik et al. 2014). The increase in Tregs will decrease the number of effector T cells to inhibit the production of CD3-induced pro-inflammatory cytokines, such as interferon (IFN)-γ and tumor necrosis factor (TNF)-α (Admyre et al. 1950).

### Strengthening the intestinal barrier

Destruction of the epithelial barrier and subsequent translocation of intestinal bacteria makes the host susceptible to NEC (Ravisankar et al. 2018). Tong et al. discovered that orally ingested BME might restore the integrity of the intestinal barrier (Tong et al. 2023). BME enhance the phosphorylation of adenosine monophosphate activated protein kinase (AMPK) and increase the secretion of the downstream protein, glucagon-like peptide-2 (GLP-2). The upregulation of AMPK and GLP-2 promotes the expression of intestinal tight junction proteins, such as zonula occludens-1 (ZO-1), claudin-1, and occludin, to protect the intestinal barrier and circumvent the invasion of pathological bacteria (Tong et al. 2023; He et al. 2021; Chiba and Maeda 2023; Dong et al. 2014).

The mucous layer is mainly composed of mucins secreted by specialized intestinal epithelial cells (IECs) and goblet cells (Pelaseyed et al. 2014), the deficiency of which had been suggested to contribute to intestinal injury in NEC. Li B et al. discovered that BME could stimulate the production of mucus by goblet cells (Li et al. 2019). Additionally, BME may facilitate the correct folding of mucins disrupted by endoplasmic reticulum stress to maintain the mucosal barrier (Miyake et al. 2020).

### Promoting cell proliferation

BME promote the development and maturation of IECs through multiple pathways (Wang et al. 2019b). Murine BME may upregulate the expression of leucine-rich repeat-containing G protein-coupled receptor 5 (Lgr5), a marker of active proliferation in intestinal stem cells (ISCs) (Hock et al. 2017). Hosfield et al. found that preterm infants with Lgr5 deficiency are associated with diminished population of ISCs and exhibit increased susceptibility to NEC (Hosfield et al. 2022). ISCs maintain IECs regeneration by modulating their proliferation and differentiation, as well as promoting intestinal development through the paracrine signaling pathways, including the hedgehog, BMP, and Notch signaling pathways (Venkatraman et al. 2021).

Exosomes derived from porcine milk promote intestinal development and IECs multiplication by increasing the expression of caudal type homeobox transcription factor 2 (CDX2), proliferating cell nuclear antigen (PCNA), and insulin-like growth factor 1 (IGF-1) receptor (Chen et al. 2016). CDX2, a gut-specific transcription factor, has been proven to be involved in the regulation of intestinal development and differentiation (Crissey et al. 2011). PCNA is related to nucleic acid metabolism and can be used as an indicator of cell proliferation (Cardano et al. 2020). The ligation of IGF-1 and IGF-1 receptors activated the downstream PI3K/AKT and MAPK/ERK signaling pathways to promote cell proliferation and differentiation (Józefiak et al. 2021).

### Alleviating cell injury

BME may alleviate ischemia and reperfusion damage, which is an important risk factor of NEC, and decrease the occurrence of short bowel syndrome and intestinal failure (Wang et al. 2022). Reduction in pro-inflammatory cytokines in the toll-like receptor 4 (TLR-4) pathways, such as TNF-α and IL-6β, was observed in mouse models pretreated with BME, which showed alleviation of intestinal inflammation (He et al. 2021).

Preterm infants are highly susceptible to oxidative stress, which is a major contributing factor of NEC (Lembo et al. 2021). Excessive oxidative system activation induces intestinal cell apoptosis and disrupts intestinal homeostasis. BME protect ISCs against oxidative stress injury in vitro by promoting the expression of Axin2, c-Myc, and Cyclin D1 in Wnt/β-catenin signaling (Dong et al. 2020). Similarly, Martin et al. also found that BME might attenuate IECs death induced by oxidative stress (Martin et al. 2018).

### Regulating genetics and metabolism

Genetic mutations have been associated with increased risk of NEC (Donda et al. 2022; Jilling et al. 2018). Interestingly, the expression of genes associated with chromosomal separation, aggregation, and mitosis was significantly increased after the administration of BME. Research has shown that BME are involved in myelination and can ameliorate NEC-related brain damage, mainly by upregulating the expression of myelin basic protein and inhibiting abnormal microglial proliferation (Hu et al. 2023). Currently, studies correlating exosomes, genetics and metabolism, and NEC are absent; therefore, more attention should be paid to the links among them.

### Protective effects of exosome contents

A proteomic analysis study of BME reported that proteins in exosomes are involved in cell growth, inflammatory signal transduction, intestinal immune system regulation, and correlated with NEC development (Herwijnen et al. 2016). Lactoferrin in BME may regulate cell survival rate and prevent NEC. In addition, the primary protein observed in human colostrum exosomes is plastin-2, which inhibits bacterial invasion and serves as a biological marker of inflammatory diseases (Yang et al. 2017; Sun et al. 2022; Li et al. 2023). Preterm and term BME exhibit distinct peptide compositions. Term BME are rich in fibrinogen gamma chain and vimentin, whereas preterm BME contain lactotransferrin, casein, and other specific peptides. These components play protective roles by maintaining the integrity of villus trophoblasts and promoting the proliferation of IECs (Wang et al. 2019b).

RNAs in BME also play a specific and important role in NEC protection. The main types of RNA that have been found to be relevant are miRNAs, and circular RNAs (circRNAs) (Melnik et al. 2021; Yan et al. 2022). Unlike proteins, miRNAs in preterm and term, or digested and undigested milk are similar, suggesting high miRNA retention in exosomes (Kahn et al. 2018). These miRNAs play important roles in the epigenetic modulation of target cells, including the cell cycle, differentiation, metabolism and apoptosis. CircRNAs are post-transcriptional regulators of gene expression and highly expressed in mammalian intestinal epithelial cells, targeting miRNAs to influence downstream cellular pathways. Many RNAs have been found to exert a series of effects, including mediating intestinal immunity and preventing cell injury, which make BME administration a potential preventive measure for NEC (Table 1).

**Table 1:** RNAs in diverse breast milk-derived exosomes in necrotizing enterocolitis (NEC)

Study	Model	RNA type	Administration method	Protective effect	Mechanism
Xie et al. (2019)	Porcine milk	miR-4334 and miR-219	Administered orally by gavage	Anti-inflammation	Inhibit TLR-4/NF-κB signaling pathways
		miR-338		Anti-apoptosis	Inhibit the p53, FAS, and Caspase-3 pathways
Guo et al. (2022)	Human breast milk	miR-148a-3p	Administered intraperitoneally	Anti-inflammation and restoration of tight junctions	Inhibit Tp53/NF-κB expression; upregulate sirtuin 1 and ZO-1 levels
Zhou et al. (2021)	Human breast milk	hsa_circRNA_405708	–	Induce the proliferation and migration of IECs	Promote VEGF protein expression
Gao et al. (2021)	Yak milk	bta-miR-34a	–	Enhance intestinal barrier	Promote IEC-6 survival
Jiang and Lönnerdal (2022)	Human breast milk	miR-22-3p	–	Promote IEC proliferation	Silence C/EBPδ gene

miR: microRNA; TLR-4: Toll-like receptor 4; NF-κB: Nuclear Factor-κB; FAS: Factor associated suicide; Tp53: Tumor protein 53; ZO-1: Zonula occludens-1; circRNAs: circular RNAs; VEGF: Vascular endothelial growth factor; IEC: Intestinal epithelial cell; C/EBPδ: CCAAT/enhancer binding proteins δ

MiRNAs that may protect against NEC have been found to be enriched in breast milk (Alexander et al. 2015; Reif et al. 2019). Xie et al. found that cotransfection of miR-4334, miR-219, and miR-338 was more effective in preventing lipopolysaccharide-induced epithelial apoptosis than transfection of any of these miRNAs alone (Xie et al. 2019). Jie’s result also indicated that miR-31 or miR-155 sponge alone did not inhibit cell growth, whereas together they could inhibit tumor cell growth (Jie et al. 2022). In addition, miR-20a/b, miR-17, miR-93 and miR-106a/b all can target p21, indicating that the combination of multiple miRNAs may have a greater impact on gene expression regulation and may overcome the limitations of individual miRNA applications (Peter 2010).

Fatty acids have been shown to reduce the incidence of NEC and modulate gastrointestinal microbial ecology a long time ago (Ran-Ressler et al. 2011). Chen et al. identified 395 lipids in human breast milk and found that most of the lipids in exosomes from term and preterm breast milk were the same, and found that lipids may significantly promote the proliferation and migration of IECs to reduce NEC severity by modulating the MAPK/ERK signaling pathway (Chen et al. 2021b). The ω-3 oxylipins in BME relieve oxidative stress and cytotoxicity in enterocytes, restore tissue integrity and prevent fibrosis (Gómez-Ferrer et al. 2023). Hence, the potential functions of lipid components in BME in preventing NEC should be investigated.

### Factors that affect BME performance

BME of mothers of preterm and preterm infants, or of different periods (colostrum or mature breast milk) can exhibit particular characteristics (Carr et al. 2021; Hata et al. 2010b). Quantitative proteomic analysis of exosomes in colostrum and mature milk has revealed that exosomes in colostrum are rich in proteins (Samuel et al. 2017; Li et al. 2024). Gao et al. also found that colostrum-derived exosomes are better at lowering inflammatory cytokine levels and protecting injured IECs than those from mature breast milk, indicating that colostrum is an optimal source of BME (Gao et al. 2019). Exosomes derived from preterm and term breast milk also vary in their components (Chen et al. 2021b; Mourtzi et al. 2021). Wang et al. demonstrated that following the co-culture of label-treated exosomes with IECs, exosomes from both preterm and term infant breast milk were internalized by IECs and localized to the cytoplasm. Notably, there was an obvious increase in the number of proliferating and migrating IECs, and the integrity of villi was significantly preserved in preterm BME-treated group compared to the term BME-treated group (Wang et al. 2019b).

Breast milk has been demonstrated to protect against NEC (Jantscher-Krenn et al. 2012; Winok et al. 2021; Elizabeth et al. 2013). However, mothers of premature infants may not be able to provide adequate milk to meet their infants’ needs, due to extrinsic factors, including inadequate breast stimulation resulting from maternal separation, severe postpartum illness, and intrinsic motivational deficits (Yaqi et al. 2023; Teresa and Gert Francois 2014). Miyake et al. found that pasteurized and fresh BME could similarly attenuate mucosal injury and inflammation caused by NEC without damaging healthy organs (Miyake et al. 2020). Thus, if self-breast milk feeding is not adequate, pasteurized donor breast milk feeding may be a feasible substitute to prevent NEC (Buckle 2017). Furthermore, stored donor breast milk has been reported to be as effective as fresh breast milk in preventing NEC, and can reduce the cost of caring for preterm infants in NICU (Johnson et al. 2020; Emma et al. 2020).

## Exosomes derived from other sources

### Stem cell-derived exosomes

Stem cell-derived exosomes may function in preventing and treating NEC (Delavogia et al. 2022a). Exosomes derived from amniotic fluid-derived mesenchymal and neural stem cells, bone marrow-derived mesenchymal stem cells, and enteric-derived neural stem cells can decrease the morbidity and severity of NEC (McCulloh et al. 2018a). Aside from the promotion of differentiation and proliferation of IECs, the therapeutic effects of stem cells mainly rely on the paracrine mechanism (Bahr et al. 2012). Stem cell-derived exosomes act through similar mechanisms and have been regarded as a cell-free alternative for tissue regeneration (McCulloh et al. 2018a). Exosomes derived from amniotic fluid-derived stem cells appear to be the main focus (Eaton et al. 2013).

Stem cell-derived exosomes are able to lower the incidence and raise the survival rate of NEC through several pathways, including the maintenance of the intestinal barrier and the transfer of IECs to accelerate injury healing (Rager et al. 2016; Wang et al. 2020; McCulloh et al. 2018b; Li et al. 2020). Extracellular vesicles from amniotic fluid stem cells can reduce intestinal inflammatory injury by activating ISCs and the Wnt signaling pathway. This mechanism is different from the mechanism by which BME inhibit inflammatory responses in that they work on the migration of IECs (Hu et al. 2023; Li et al. 2020). Premature infants and those prone to developing NEC early in life appear to suffer from metabolic dysfunction (Sinclair et al. 2020). Hu et al. reported that exosomes derived from amniotic fluid stem cells could regulate hemostasis, promote the reabsorption of calcium and sodium, as well as restore the production of immunoglobulin A in the intestines of mice with NEC (Hu et al. 2023).

### IECs-derived exosomes

IECs can secrete exosomes from the apical and basolateral sides, which contains major histocompatibility complexes I and II, CD63, CD26/dipeptidyl-peptidase IV, and A33 antigen. And the increased production of these exosomes may happen in response to oxidative stress (Niel et al. 2001). IECs-derived exosomes are involved in the development of intestinal diseases and therefore, have the potential to be used as biomarkers of NEC. IECs-derived exosomes contain ribosomal proteins, which are involved in intestinal homeostasis, inflammation, and immune responses (Ding et al. 2022). Furthermore, IECs-derived exosomes can independently process and present antigens to the intestine (Lin et al. 2005). Exosomes rich in transforming growth factor-β1 and IL-10 stimulate the immunosuppression of dendritic cells and Tregs and play an important role in intestinal immune tolerance to reduce inflammation (Jiang et al. 2016).

IECs-derived exosomes can carry miRNAs to local or distant immune cells and regulate inflammation (Park et al. 2022). For example, exosomes transporting miR-23a-3p alleviate gut ischemia/reperfusion damage by targeting at MAP4K4 (Yang et al. 2022). Exosomes transporting miR-29a secrete IFN-γ, IL-6, and IL-1β to impair epithelial integrity by suppressing the expression of tight junctions (Appiah et al. 2020). The function of IECs-derived exosomes depends on the type of cargo that they carry; therefore, the selection of effective exosome species might be crucial to its application in the treatment of NEC.

### Plant-derived exosomes

Certain plants, such as grapes and grapefruits, can also secrete exosome-like nanoparticles, and have been proven to alleviate the severity of intestinal injury in a dextran sulphate sodium-induced colitis animal model (Kim et al. 2022; Nemati et al. 2022). These exosome-like nanoparticles work on immune cells in the gut, such as dendritic cells, monocytes, and macrophages. They can regulate inflammatory cytokines and chemokines, and play a role in intestinal immune regulation (Wang et al. 2014; Deng et al. 2017; Liu et al. 2022; Sriwastva et al. 2022). These nanoparticles may suppress the expression of pro-inflammatory factors including TNF-α, IL-1β, and CD11b, enhance the expression of antioxidant gene, heme oxygenase-1, and increase the expression of anti-inflammatory cytokines, such as IFN-γ and IL-10, by modulating the AMPK, NF-κB, and AhR-COPS8 signaling pathways, respectively (Wang et al. 2014; Deng et al. 2017; Liu et al. 2022; Sriwastva et al. 2022).

When exosome-like nanoparticles derived from grapes are taken up orally and absorbed by intestinal cells, they may activate the Wnt/β-catenin signaling pathway to promote the proliferation of the ISCs marked by Lgr5, and thereby maintain intestinal homeostatic renewal and differentiation to IECs (Stewart et al. 2021; Ju et al. 2013). Exosome-like nanoparticles derived from ginger can be taken up by *Lactobacillus rhamnosus* in the gut, which influences intestinal flora and promotes the growth of *L. rhamnosus*. Intestinal tissue exposed to these nanoparticles activates AhR to induce the production of IL-22, a key substance that maintains the intestinal barrier and the inhibition of TNF-α and IL-1β (Teng et al. 2018).

### Bacteria-derived exosomes

More than 100 trillion microorganisms inhabit the human gut and interact with the host in a symbiotic relationship to regulate host immune system development (Rinninella et al. 2019). The acquisition and composition of the gut microbiota during infancy affect human health throughout life (Zeng et al. 2022; Pantazi et al. 2023). Gut microbiota plays an important role in the development of NEC (Moschino et al. 2022). Gut microbiota-derived exosomes can interact with immune cells to regulate inflammatory responses (Macia et al. 2019). Bacteria-derived exosomes in breast milk act as vectors to transport bioactive molecules or move themselves to host cells, affecting intestinal colonization and immunity of the infant (Gao et al. 2018; Kim and Yi 2020).

**Fig. 3:**
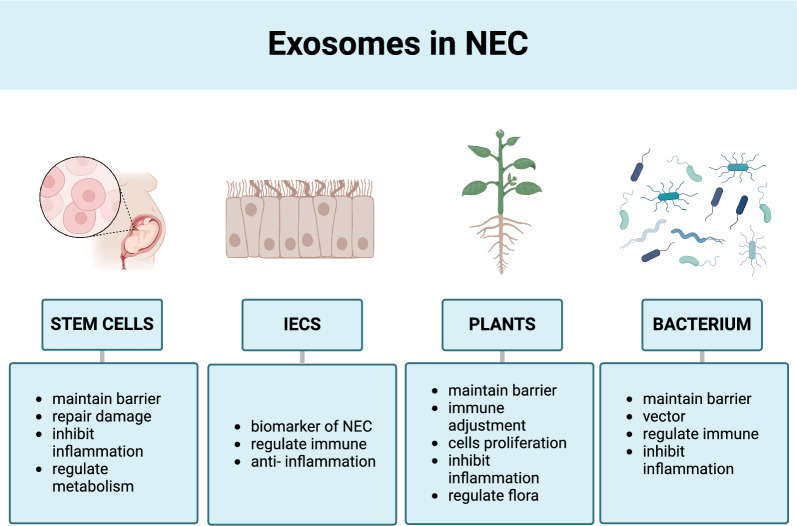
Exosomes derived from other sources and their protective effects against NEC. Exosomes can be extracted from stem cells, IECs, plants, and bacteria, in addition to being derived from breast milk. As shown in the figure, these exosome sources have also been shown to be beneficial for NEC treatment (Figure created with BioRender.com)

Bacteria-derived exosomes enhance the barrier function of IECs by stimulating the expression of tight junction proteins, upregulating the expression of endothelial cell adhesion molecules, and promoting antigen presentation. Natural nanocarriers for immunogenic antigens can enter systemic circulation to induce various immune and metabolic responses (Yu et al. 2018). However, as cargo carriers, bacteria-derived exosomes can also transport toxins to host cells, causing cell damage and inducing excessive local inflammation and intestinal flora imbalance. Hence, targeted intervention for the release of exosomes from intestinal flora to maintain the dynamic balance between the host and gut bacteria may prevent the occurrence of NEC (Liu et al. 2021) (Fig. 3).

## Application and current obstacles of BME in NEC

Current research found that stem cells and breast milk-derived exosomes mainly exert their therapeutic effects through paracrine pathways (Hongtao et al. 2020; Shome et al. 2021). The unique lipid bilayer structure in exosomes protects the contents from RNA enzymes and proteases, so that exosomes have significant advantages over other vehicles as natural carriers of endogenous biomolecules (Pozo-Acebo, et al. 2021; Kim et al. 2020; Silva et al. 2021). BME are extremely stable under conditions like low pH, boiling, and freezing; they can preserve the bioavailability of their content and be used for oral administration of drugs that used to be given intravenously (Betker et al. 2019; Carobolante et al. 2020).

BME exhibit superior biocompatibility and a long half-life. They can cross the intestinal mucosa, the placenta and the blood–brain barrier, thus enabling the delivery of therapeutic agents to hard-to-reach tissue (Las et al. 2022; Ngu et al. 2023). BME can also function in neurological development (Zhou et al. 2022), the endocrine/metabolism (Zempleni et al. 2019), the cardiovascular system (Meng et al. 2024), and bone remodelling (Hao et al. 2024), indicating a promising future of BME-based therapy.

At present, studies on the feasibility and safety of BME application are lacking. Separation and purification techniques for cost-effect efficient yields are also limited. Finding a separation technique to ensure the sufficient quantity and purity of exosomes remains challenging (Wijenayake et al. 2021; Weiskirchen et al. 2023). Biological effects vary depending on the processing procedures, including ultra-centrifugation and isoelectric precipitation (Yamauchi et al. 2019; Kleinjan et al. 2021). All current separation methods have disadvantages and may introduce a certain proportion of contaminants (Tauro et al. 2012).

Like liposomes, BME have a dual lipid membrane and an aqueous core; therefore, they can potentially be loaded with both hydrophilic and lipophilic drugs (Vlassov et al. 2012). The existing loading methods include electroporation (Mirzaei et al. 2023), ultrasonication (Luo et al. 2021), and nanofluids (Hao et al. 2021). However, no conclusion has been reached regarding the specific dose, frequency, or route of administration (Munagala et al. 2016).

A statement in 2015 expressed the feasibility of clinical application of exosomes, and emphasized the need for safety and relevant laws regulating their production and clinical applications (Lener et al. 2015). In addition, in vitro uptake mode, markers, and storage remain yet to be overcome. In a word, much remains to be developed regarding the study of exosomes, particularly in the field of BME.

## Conclusions

While there is a general awareness of NEC, effective strategies to prevent its incidence, improve long-term outcome and reduce mortality remain elusive. Exosomes, especially BME show significant potential as a cell-free therapy for NEC, attracting considerable interest from researchers. Cell-based therapies in neonates may present more challenges and risks compared to those in adults due to their young age, limited number of experimental studies and ethical considerations (Delavogia et al. 2022b). In contrast, BME offer significant advantages, including easy availability, low immunogenicity, and high absorption rate, making them a promising alternative in the treatment of NEC (Pisano et al. 2020).

Although some therapeutic mechanisms of BME against NEC have been discovered (Hu et al. 2023; Li et al. 2019; Hock et al. 2017; Pacella et al. 2023; Tong et al. 2023; Chen et al. 2016; Miyake et al. 2020; Dong et al. 2020), more mechanisms and therapeutic approaches are still being investigated. Further research is essential to elucidate the underlying mechanisms and address potential side effects associated with BME. This deeper understanding will be crucial for facilitating effective clinical advancements.

## Data Availability

Not applicable.

## Abbreviations

NEC: Necrotizing enterocolitis
NICU: Neonatal intensive care unit
miR: MicroRNA
BME: Breast milk-derived exosomes
Tregs: Regulatory T cells
IECs: Intestinal epithelial cells
IL: Interleukin
AMPK: Adenosine monophosphate-activated protein kinase
GLP-2: Glucagon-like peptide-2
Lgr5: Leucine-rich repeat-containing G protein-coupled receptor 5
ISCs: Intestinal stem cells
CDX2: Caudal type homeobox transcription factor 2
PCNA: Proliferating cell nuclear antigen
IGF-1: Insulin-like growth factor 1
IFN: Interferon
TNF: Tumor necrosis factor
TLR-4: Toll-like receptor 4
PI3K/AKT: Phosphatidylinositol 3 kinase/protein kinase B
MAPK/ERK: Mitogen-activated protein kinase/extracellular regulated protein kinases
circRNAs: Circular RNAs
